# Copy number variation in the porcine genome inferred from a 60 k SNP BeadChip

**DOI:** 10.1186/1471-2164-11-593

**Published:** 2010-10-22

**Authors:** Yuliaxis Ramayo-Caldas, Anna Castelló, Romi N Pena, Estefania Alves, Anna Mercadé, Carla A Souza, Ana I Fernández, Miguel Perez-Enciso, Josep M Folch

**Affiliations:** 1 Departament de Ciència Animal i dels Aliments, Facultat de Veterinària, Universitat Autònoma de Barcelona, 08193 Bellaterra, Spain; 2 Genètica i Millora Animal, IRTA Lleida, 25198 Lleida, Spain; 3 Departamento de Mejora Animal SGIT-INIA, 28040 Madrid, Spain; 4 Institut Català de Recerca i Estudis Avançats (ICREA), Barcelona, Spain; 5 Department of Animal Production, University of Lleida, 25198 Lleida, Spain; 6 Universidade Católica de Brasília, Brazil

## Abstract

**Background:**

Recent studies in pigs have detected copy number variants (CNVs) using the Comparative Genomic Hybridization technique in arrays designed to cover specific porcine chromosomes. The goal of this study was to identify CNV regions (CNVRs) in swine species based on whole genome SNP genotyping chips.

**Results:**

We used predictions from three different programs (cnvPartition, PennCNV and GADA) to analyze data from the Porcine SNP60 BeadChip. A total of 49 CNVRs were identified in 55 animals from an Iberian x Landrace cross (IBMAP) according to three criteria: detected in at least two animals, contained three or more consecutive SNPs and recalled by at least two programs. Mendelian inheritance of CNVRs was confirmed in animals belonging to several generations of the IBMAP cross. Subsequently, a segregation analysis of these CNVRs was performed in 372 additional animals from the IBMAP cross and its distribution was studied in 133 unrelated pig samples from different geographical origins. Five out of seven analyzed CNVRs were validated by real time quantitative PCR, some of which coincide with well known examples of CNVs conserved across mammalian species.

**Conclusions:**

Our results illustrate the usefulness of Porcine SNP60 BeadChip to detect CNVRs and show that structural variants can not be neglected when studying the genetic variability in this species.

## Background

The pig (*Sus scrofa*) is one of the most widespread livestock species and one of the most economically important worldwide. The porcine genome has a total of 18 autosomes plus the X/Y sex chromosome pair; it is similar in size, complexity and chromosomal organization to the human genome. In contrast to SNPs and microsatellites, structural variations have received considerably less attention in pigs. Copy number variants (CNVs) are DNA segments ranging in length from kilobases to several megabases with a variable number of repeats among individuals [1]. Segmental duplications and CNVs have been extensively studied in other organisms [2-7]. Previous studies at genome scale suggest that CNVs comprise 5-12% of the human and ~4% of the dog genome [5,8-10]. CNVs can influence gene expression, affect several metabolic traits and have been associated with Mendelian and complex genetic disorders [1].

Recent studies in pigs have detected CNVs using the Comparative Genomic Hybridization (CGH) technique in arrays designed to cover specific porcine chromosomes [11,12]. An alternative, cheaper method for CNV detection is based on whole genome SNP genotyping chips [13-15], but it has not been tested yet, to our knowledge, in the swine species. A high-density porcine SNP BeadChip has recently been released by *Illumina*, which contains probes to genotype 62,163 SNPs covering the whole genome. This platform has an average distance between SNPs of 39.61 kb in autosomes and 81.28 kb in chromosome X (based on Sscrofa9 genome sequence assembly) and is a very valuable resource to study pig genetic variability and the molecular dissection of complex traits of economic importance [16].

The goal of this study was to detect CNV regions (CNVRs) from the Porcine SNP60 BeadChip data on autosomal chromosomes using a pedigree from an Iberian x Landrace (IBMAP) cross and to validate them in a collection of unrelated pigs from different origins.

## Results and Discussion

### Detection of structural variants

**Figure 1:**
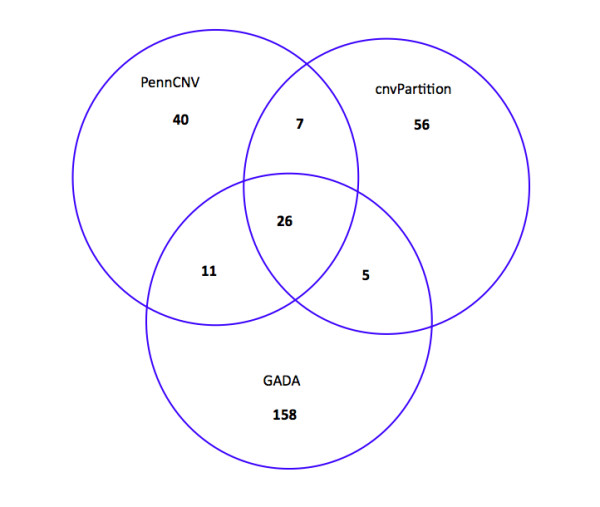
**Overlapping CNV events from the three programs used in the analysis**.

The Porcine SNP60 BeadChip data from 55 IBMAP animals were analyzed by multiple predictions from three different programs: cnvPartition (Illumina), PennCNV [17] and GADA [18]. The initial number of CNVs called by each software was 94, 84, and 200, respectively. Figure 1 summarizes the CNVs identified and compares the results obtained from the three programs.

**Figure 2:**
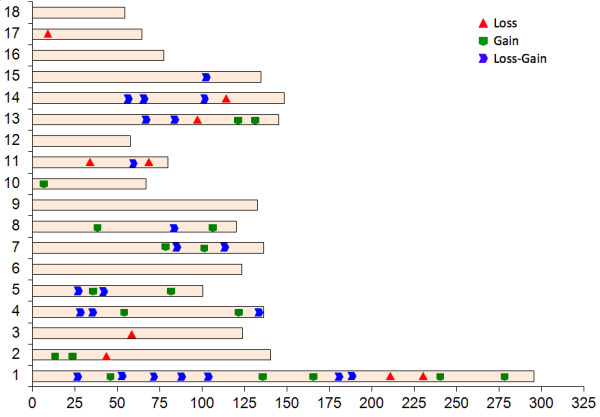
**Graphical representation of the CNVRs detected**. Red triangles represent loss predicted status, gains are indicated in green and regions with either loss or gain status are represented in blue. X-axis values are chromosome position in Mb. Y-axis values are chromosome name. Chromosome sizes are represented in proportion to real size of the *Sus scrofa *karyotype obtained from the ENSEMBL data base.

For further analyses, we retained only CNVs applying a more stringent criterion, namely CNV regions (CNVRs) containing overlapping CNVs recalled by at least two programs, spanning three or more consecutive SNPs and detected in a minimum of two animals. A total of 49 CNVRs located in 13 of the 18 analyzed autosomal chromosomes were identified (Figure 2). All of these CNVRs showed Mendelian inheritance in animals across several generations of the IBMAP cross and therefore are unlikely to be artefacts or false positives, suggesting that our empirical criterion to retain CNVRs is reasonable.

The percentage of CNVRs confirmed by at least two programs was 52.38% for PennCNV, 21% for GADA and 40.42% for cnvPartition. A total of 26 CNVRs (53.06%) were detected by the three algorithms (Figure 1). Similar results were reported by Winchester et al. (2009) comparing different algorithms for CNV detection, suggesting that PennCNV is the most accurate program in the prediction of CNVs for the Illumina's platform [19]. In a recent study [20], the relative performance of seven methods for CNV identification was evaluated showing that the PennCNV algorithm has a moderate power and the lowest false positive rate. This is likely explained by the unique ability of this algorithm to integrate family relationships and signal intensities from parent-offspring trios data. The low percentage of CNVs called by the GADA software might be explained by the relative low coverage of the Porcine SNP60 BeadChip.

The size of the CNVRs detected ranged from 44.7 kb to 10.7 Mb, with a median size of 754.6 kb (Table 1). The Porcine SNP60 BeadChip was originally developed for high-throughput SNP genotyping in association studies. Although CNV detection is feasible with this technology, it is impaired by low marker density, non-uniform distribution of SNPs along pig chromosomes and lack of non-polymorphic probes specifically designed for CNV identification [16]. Hence, only the largest CNVRs are expected to be assessed with the Porcine SNP60 BeadChip. This explains the difference in minimum CNV length between our study (44.7 kb) and the work of Fadista et al., 2008 (9.3 kb) using the CGH technique.

**Table 1:** Description of the 49 CNVRs detected in the pig genome

CNVR ID	**Chr**.	Start	End	Length (Kb)	Status	Number of Animals
1	1	24217581	24340263	122.68	Loss-Gain	13
2	1	45428275	45549702	121.43	Gain	7
3	1	48467320	48606417	139.10	Loss-Gain	24
4	1	65873816	72839149	6965.33	Loss-Gain	50
5	1	84499444	86580142	2080.70	Loss-Gain	101
6	1	102184641	102246547	61.91	Loss-Gain	73
7	1	160653704	161917767	1264.06	Gain	49
8	1	179815713	179914790	99.08	Loss-Gain	197
9	1	181518754	181741363	222.61	Loss-Gain	86
10	1	206491920	206869145	377.23	Loss	12
11	1	222291368	233007189	10715.82	Loss	11
12	1	237242221	237929823	687.60	Gain	8
13	2	11601476	11714214	112.74	Gain	5
14	2	21970642	22174716	204.07	Gain	7
15	2	40533655	41466383	932.73	Loss	270
16	3	56202930	56347970	145.04	Gain	9
17	4	24971805	25077329	105.52	Loss-Gain	39
18	4	33537533	33962195	424.66	Loss-Gain	8
19	4	50569393	51322987	753.59	Gain	14
20	4	119210502	119256548	46.05	Gain	7
21	4	134367793	134519459	151.67	Loss-Gain	21
22	5	23899971	24070933	170.96	Loss-Gain	51
23	5	35733150	37322403	1589.25	Loss-Gain	51
24	5	35933178	36136940	203.76	Gain	12
25	7	77371678	77536074	164.40	Gain	18
26	7	82665585	83053927	388.34	Loss-Gain	18
27	7	97896821	98115996	219.18	Gain	21
28	7	110105319	110156658	51.34	Loss-Gain	11
29	10	4479233	4701713	222.48	Gain	42
30	11	65309203	65381195	71.99	Loss	20
31	11	56381032	57812846	1431.81	Loss-Gain	45
32	13	64352825	64798051	445.23	Loss-Gain	105
33	13	118821556	118923746	102.19	Gain	26
34	14	53301700	55310453	2008.75	Loss-Gain	10
35	14	63834660	63882223	47.56	Loss-Gain	176
36	14	111363926	111540634	176.71	Loss	150
37	14	97526178	99696847	2170.67	Loss-Gain	49
38	15	99843606	100116097	272.49	Loss-Gain	75
39	17	6525429	6635237	109.81	Loss	84
40	8	80936785	81030481	93.70	Loss-Gain	93
41	13	81503361	81601546	98.19	Loss-Gain	118
42	1	130025060	130085466	60.41	Gain	26
43	1	276666775	276786004	119.23	Gain	13
44	5	79779580	79931050	151.47	Gain	34
45	8	35691571	36104826	413.26	Gain	53
46	8	104917016	104968103	51.09	Gain	5
47	11	30072771	30117419	44.65	Loss	38
48	13	94769297	94922644	153.35	Loss	66
49	13	128150975	128360061	209.09	Gain	17

The genomic coordinates are expressed in bp and are relative to the *Sus scrofa *April 2009 genome sequence assembly (Sscrofa9)

Among the first 55 animals analyzed, a single CNVR (*CNVR35*) was called in two animals whereas the remaining 48 CNVRs were called in three or more animals. A segregation analysis was performed in 372 additional animals from the IBMAP cross and the distribution of the CNVRs was additionally studied in 133 unrelated pig samples from different geographical origins (see Methods). All initially detected 49 CNVRs were segregating in the IBMAP cross and 41 were also detected in American pig populations (Additional file 1, Table S1). The number of animals with alternative alleles for the CNVRs ranged from five (*CNVR13, CNVR46*) to 270 (*CNVR15*). The predicted status for the CNVRs was 19 (38.7%) for gain, eight (16.3%) for loss and 22 (45%) for regions with gain or loss status in different animals (Table 1). This proportion may be related to natural selection, as it is assumed that the genome is more tolerant to duplications than to deletions [21-24]. The high percentage of CNVRs with gain or loss status may be the result of including in the analysis pig breeds with different genetic origins and from different countries. However, to establish the real status of CNVRs, validation by other techniques such as quantitative PCR (qPCR) will be necessary.

### Genes located within CNVRs

The Biomart software in the Ensembl Sscrofa9 Database was used to retrieve genes annotated within the genomic regions of CNVRs. A total of 153 protein-coding genes, four miRNA, six miscRNA, three pseudogenes, two rRNA, two snoRNA and nine snRNA were annotated within the 49 CNVRs (Additional file 2, Table S2). Two or more annotated genes were found in 15 CNVRs, whereas one gene only was located in 14 CNVRs. No annotated genes were identified in 20 CNVRs, but this can be due to the incomplete annotation of the Sscrofa9 genome sequence assembly. In contrast to the high number of genes found in this study, it has been suggested that CNVs are located preferably in gene-poor regions [25,26], probably because CNVs present in gene-rich regions may be deleterious and therefore removed by purifying selection [24].

### Validation by quantitative PCR

Real time quantitative assays were designed for CNVR validation on seven genomic regions simultaneously detected with the three programs (CNVRs 1, 3, 15, 17, 22, 32, and 36; Table 1). Five of these CNVRs (15, 17, 22, 32, and 36) were confirmed by qPCR, nevertheless fewer animals were validated for CNVRs 15, 17, and 32 (Additional file 3, Fig. S1). Thus, the false discovery rate (FDR) for the seven analyzed CNVRs was 29%; it should be noted that the percentage of CNVRs validated in this study (71%) is higher than previously reported in pigs (50%) [11]. This result might be explained by the stringent criteria used in our analysis, which was proposed in order to increase confidence and minimize the false positives. Nevertheless, we were not able to confirm two of the CNVRs..Several factors may account for the discrepancy in CNVR prediction between the *in silico *analysis of Porcine SNP60 BeadChip data and the qPCR method. First, the incomplete status of the 4× sequence depth Sscrofa9 assembly and the low probe density of the Porcine SNP60 BeadChip makes it difficult to establish the true boundaries of CNVRs and may result in an over estimation of their real size. Therefore, it cannot be ruled out that the primers used to validate the CNVRs by qPCR may have been designed outside the structural polymorphic region. Second, polymorphisms such as SNPs and indels may influence the hybridization of the qPCR primers, changing the relative quantification (RQ) values for some animals. Finally, the true CNVR boundaries may be also polymorphic between the analyzed animals.

**Figure 3:**
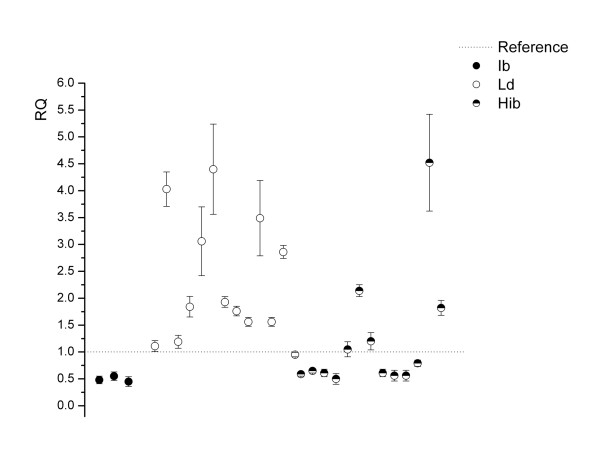
**Analysis by quantitative PCR (qPCR) of *CNVR36 *(*CYP4502 C32 Fragment *gene)**. Twenty-one animals with statistical evidence for CNVR and eight false negative animals from the control group are showed. The horizontal dashed line represents the relative quantification (RQ) value of the reference animal. Each dot represents the relative copy number in comparison to the reference individual. Y-axis shows the RQ value obtained by qPCR. Vertical bars represent the standard error. Breed abbreviations are: **Ib**: Iberian; **Ld**: Landrace; **Hib**: animals belonging to several generations of the IBMAP cross (F1, F2, and BC); **Mx**: Mexican hairless; **Brz**: Brazilian local breed; **Gu**: Guatemala local breed; **Yu**: Yucatan miniature pig, **CC**: Cuban creole pig.

For the qPCR validation of *CNVR36*, a PCR protocol for the *Cytochrome P4502 C32 Fragment gene *[EMBL: ENSSSCG00000010487] was designed. A total of 37 animals were analyzed: 21 with statistical evidence for CNVR and 16 without the CNVR (control group). One of the animals from the control group was selected as reference. Six false positive animals were observed, indicating a FDR of 29% for CNVR36 (Figure 3).

**Figure 4:**
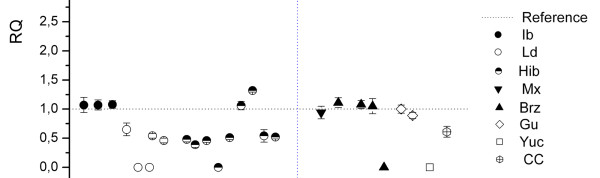
**Analysis by quantitative PCR (qPCR) of *CNVR22 *(*SLC16A7 *gene)**. Twenty-one animals with statistical evidence for CNVR, three false negative and two Iberian from the control group are plotted. The horizontal dashed line represents the relative quantification (RQ) value of the reference animal. Each dot represents the relative copy number of the animal in comparison to the reference. Y-axis shows the RQ value obtained by qPCR. Vertical bars represent the standard error. The vertical dashed line separates the 17 related IBMAP individuals from the nine unrelated American local breeds. Breed abbreviations as described in Figure 3.

A qPCR assay with primers located in the *SLC16A7 *gene [EMBL: ENSSSCG00000000456] was used for *CNVR22 *validation. A total of 50 animals were analyzed: 21 with statistical evidence for CNVR (12 from the IBMAP cross and nine unrelated individuals belonging to six different breeds of American populations) and 29 without the CNVR (control group). One of the animals from the control group was selected as reference. Nine of the IBMAP cross animals were validated by qPCR (FDR = 25%). Conversely, only three animals from the American populations were validated by qPCR, suggesting a higher FDR (67%) (Figure 4). These differences in FDR may be explained by the higher accuracy of the PennCNV algorithm when family information is available and stress the usefulness of including family information in CNV detection. However, this conclusion should be taken with caution due to the limited number of animals analyzed.

For CNVRs 22 and 36, copy number changes were also identified by qPCR in animals where CNVs were not detected initially in the statistical analysis (three and eight animals, respectively). This represents a false negative rate of 10% (3/29) for *CNVR22 *and 50% (8/16) for *CNVR36*. The three false negative animals for *CNVR22 *were classified as deletions by qPCR protocol. A similar situation, but with a different copy number status, was observed for *CNVR36*, where the eight false negative animals showed a duplication pattern by qPCR. False negative identification is common in CNV detection, and has been reported previously using the CGH technique in pigs and other mammalian species [5,11].

Three of the validated CNVRs (17, 22, and 36) showed differential patterns of copy number variants between breeds. For instance, *CNVR22 *showed a loss (deletion) in Landrace and in animals from other breeds (Figure 4). Assuming that Iberian pigs have two copies of *CNVR22 *(qPCR RQ = 1), five animals showing an RQ = 0 by qPCR are predicted to be homozygous for a deletion on this genomic region. In *CNVR36*, a loss was found in Iberian pigs relative to Landrace animals (Figure 3). In agreement with the Mendelian segregation of this CNVR, hybrid animals show intermediate RQ values. The RQ mean values were 0.49 for Iberian, 2.51 for Landrace and 1.2 for hybrid animals.

*CNVR36 *contains a miRNA gene [EMBL: ENSSSCG00000019484] and the Cytochrome *P4502 C32 Fragment *gene (Additional file 2, Table S2), which is a member of the *Cytochrome P450 *gene family (*CYTP45O*). Proteins coded by this gene family constitute the major catalytic component of the liver mixed-function oxidase system and play a pivotal role in the metabolism of many endogenous and exogenous compounds [27]. Interestingly, CNVs comprising genes of the *CYTP45O *family have been described in humans and dogs [5,10,28], but had not been previously reported in pigs. In humans, copy number variations of *CYTP45O *genes have been associated with variation in drug metabolism phenotypes [29-31]. Differential expression of genes of the *CYTP450 *family has been correlated with androsterone levels in pigs from Duroc and Landrace breeds [32]. It has also been demonstrated that the total *CYTP450 *activity was slightly higher in minipigs compared to conventional pigs [33]. *CNVR36 *lays close to the peak position of a QTL for androsterone leves described in a cross between Large White and Chinese Meishan [34]. This suggests a possible role of this structural variation in determining androsterone levels; however, more studies will be necessary to validate this hypothesis.

*CNVR22*, also validated by qPCR, comprises the *SLC16A7 *gene. This gene belongs to the *solute carrier family 16 *gene family, which encodes 14 proteins that are largely known as monocarboxylate transporters (MCTs). The human *SLC16A7 *gene encodes the MCT2 protein [35] and it is expressed in several normal human tissues. In pigs, MCT2 may function as a housekeeping lactate transporter, regulating the acidification of glycolytic muscles [36]. Remarkably, *CNVR22 *is located in the middle of the confidence interval of a QTL for meat pH described in four pig populations [37].

Duplication events have also been validated by qPCR for *SOX14 *[EMBL: ENSSSCG00000011656] (*CNVR32*) and *INSC *[EMBL: ENSSSCG00000013385] *(CNVR15). *Copy number changes have not been previously reported in either of them in pigs. *SOX14 *is a member of the *SOX *gene family [38] of transcription factors involved in the regulation of embryo development and cell fate determination. *SOX14 *may have a major role in the regulation of nervous system development and it is a mediator of the neuronal death process [39]. *SOX14 *is an intronless gene that may has arisen by duplication from an ancestral SOX B gene, which likely was the product of a retrotransposition event [40]. *Inscuteable *(*INSC*) was first described in *Drosophila *and it plays a central role in the molecular machinery for mitotic spindle orientation and regulates cell polarity for asymmetric division [41,42]. *Inscuteable homologs *have been found in several species, including vertebrates and insects [43]. In mammals, INSC is functionally conserved and it is required for correct orientation of the mitotic spindle in retina [43] and skin [44] precursor cells.

The qPCR assay for *CNVR17 *validation was designed over the sequence of one expressed sequence tag [EMBL: EW037329]. From four Cuban creole pigs tested, three animals showed a deletion and one animal a duplication event (Additional file 3, Fig. S1).

### Other relevant CNVRs

Although other CNVRs have not been analyzed by qPCR, there is evidence of structural polymorphism in the literature. For instance, *CNVR45 *contains the *KIT *gene, a well-characterized and functionally important CNV in pigs. The dominant white coat phenotype in pigs is caused by *KIT *gene duplication or triplication and a splice mutation in one of the *KIT *gene copies [45-49]. In addition, studies in other mammals [5,6,50-55] have described CNVRs overlapping other gene families including: *Olfactory receptor family, Glutamate receptor family, Solute carrier family, Cytochrome P450 family, Cyclic nucleotide phosphodiesterases family and Fucosyltransferase family*. Twelve of the CNVRs detected in our study include or overlap porcine orthologues of these genes. Furthermore, 13 of the detected CNVRs include 47 genes previously reported in the Human Database of Genomic Variants http://projects.tcag.ca/variation/?source=hg19[56] (Additional file 4, Table S3).

## Conclusions

We have described the first CNVRs in swine based on whole genome SNP genotyping chips. A total of 49 CNVRs were identified in 13 autosomal chromosomes. These CNVRs showed Mendelian inheritance across 427 individuals belonging to several generations of an Iberian x Landrace cross, and were also confirmed in different pig breeds. Five out of seven selected CNVRs were validated by qPCR; among the remaining CNVRs we found well known examples of CNVs conserved across mammalian species. Although these results illustrate the usefulness of Porcine SNP60 BeadChip to detect CNVRs, the number detected here is probably a gross underestimate given the wide interval between SNPs in the Porcine 60 k BeadChip.

## Methods

### Animal Material

We analyzed a total of 560 animals, including 427 individuals (150 males and 277 females) belonging to several generations of the IBMAP cross. This population was originated by crossing three Iberian (Guadyerbas line) boars with 31 Landrace sows [57,58] (Additional file 5, Fig. S2). The remaining 133 pig samples were obtained from different geographical origins: 127 from American local breeds and village pigs [59], four black Sicilian pigs, one Hungarian Mangalitza and one Chinese Wild boar (Additional file 6, Table S4). We adhered to our national and institutional guidelines for the ethical use and treatment of animals in experiments.

### Genotyping

All 560 animals were genotyped with the Porcine SNP60 BeadChip (Illumina Inc., USA) using the *Infinium HD Assay Ultra *protocol (Illumina). Raw data had high-genotyping quality (call rate >0.99) and were visualized and analyzed with the GenomeStudio software (Illumina). For subsequent data analysis, a subset of 50.572 SNPs was selected by removing the SNPs located in sex chromosomes and those not mapped in the Sscrofa9 assembly.

### Statistical analysis

Following the recommendations of Winchester et al. (2009) to increase the confidence in CNV detection and limit the number of false positives, we used predictions from multiple programs. First, we used the Illumina's proprietary software GenomeStudio to check data quality and the cnvPartition v2.4.4 Analysis Plug-in for CNV detection. The minimum probe count employed was three and the remaining parameters were used according to the default criteria provided (Illumina). Then, we exported the signal intensity data of logRratio and B allele frequency to employ the R package for Genome Alteration Detection Algorithm (GADA) [18], which includes one algorithm based in sparse Bayesian learning to predict CNV changes. The multiple array analysis option was employed and the parameters defined for the Bayesian learning model and the backward elimination (BE) were: 0.8 for sparseness hyperparameter (a_α_), 8 for critical value of the BE and 3 as the minimum number of SNPs at each segment.

Next, we used the command line version of PennCNV software that integrates, in a joint-calling algorithm, a Hidden Markov Model (HMM) with family relationships, signal intensities for parent-offspring trios, marker distance and population frequency of allele B [17]. The CNV calling was performed using the default parameters of the HMM model with 0.01 of UF factor. The "-trio" and "-quartet" arguments were employed to make use of our family information.

It is unclear with this kind of data, where the statistical properties of the methods are unknown, which is the optimum strategy to balance false positives and power. Here we chose to follow a pragmatic approach, requiring that the CNV was called by at least two algorithms, detected in at least two animals and contained three or more consecutive SNPs. Hence, these genomic regions should be referred as copy number variable regions (CNVRs). To define the size of each CNVR in the genome, we used the overlapping region between CNV predictions from different programs.

Pipeline analysis for CNVR detection was initially performed in 55 individuals of the IBMAP cross (13 males and 42 females), including all founder Iberian boars (three males), 24 founder Landrace sows, 17 F1, three F2, and eight backcross animals. Subsequently, we tested the segregation of these initially detected CNVRs in the rest of the IBMAP cross animals (372), and described their distribution in 127 unrelated pig samples from American local pigs, four black Sicilian pigs, one Hungarian Mangalitza and one Chinese Wild boar.

Gene annotation within the CNVRs was retrieved from the Ensembl Genes 57 Database using the Biomart [http://www.biomart.org] software.

### Quantitative real time PCR

Quantitative real time PCR (qPCR) was used to validate seven genomic regions detected by the three methods and representing different predicted status of copy numbers. We used the 2^-ΔΔCt ^method for relative quantification (RQ) of CNVs [5,60,61]. This comparative method uses a target assay for the DNA segment being interrogated for copy number variation and a reference assay for an internal control segment, which is normally a known single copy gene; moreover a reference sample is included. The method requires the target and reference PCR efficiencies to be nearly to equal. Experiments were performed on the test and control primers to verify comparable efficiency in amplification prior to analysis of copy number.

CNVRs were quantified using the Taqman chemistry in an ABI PRISM^® ^7900HT instrument (Applied Biosystems, Inc., Foster City, CA); results were analyzed with the SDS software (Applied Biosystems). Primers and hydrolysis probes (Taqman-MGB labeled with FAM) were designed for the seven CNVR regions with the Primer Express software (Applied Biosystems). A previously described [62] design on the *glucagon *gene [EMBL:GCG] was used as single copy control region, but a single nucleotide substitution on primer forward was introduced to adapt the primer to the porcine species. Primers and probes are shown in Additional file 7, Table S5.

PCR amplifications were performed in a total volume of 20 μl containing 10 ng of genomic DNA. Taqman PCR Universal Master Mix (Applied Biosystems) was used in all reactions except in GCG amplifications, where TaqMan^® ^PCR Core Reagents (Applied Biosystems) with 2.5 mM MgCl_2 _were utilized. All primers and probes were used at 900 nM and 250 nM respectively, except *CytochromeP450 2C32 Fragment *forward primer, which was used at 300 nM. Each sample was analyzed in triplicate. The thermal cycle was: 2 min at 50°C, 10 min at 95°C and 40 cycles of 15 sec at 95°C and 1 min at 60°C. One sample without copy number variation for each of the genomic regions analyzed was used as reference.

### Data availability

The full data set have been submitted to dbVAR [63] under the accession number nstd44.

## Abbreviations

**CNV**: copy number variation; **CNVR**: CNV region; **PCR**: polymerase chain reaction; **IBMAP**: Iberian x Landrace intercross; **qPCR**: quantitative real time PCR; **RQ**: relative quantification value; ***CYTP450: ****Cytochrome P450 *gene family; ***SLC16A7: ***solute carrier family 16 member 7; ***MCT2: ***monocarboxylic acid transporter 2; ***SOX14: ***SRY (sex determining region Y)-box 14; ***INSC: ***inscuteable homolog (Drosophila).

## Authors' contributions

YRC, AIF, MPE and JMF conceived and designed the experiment. JMF was the principal investigator of the project. YRC performed the data analysis and drafted the manuscript. EA, RNP, AIF, CAS, YRC, MPE and JMF collected samples. RNP and CAS performed DNA isolation. AM and AC performed the SNP genotyping and AC did the qPCR and RT-PCR assays. All authors read and approved the final manuscript.

## Supplementary Material

Additional file 1 **Table S1**. Results of the distribution analysis in American pig populations. Click here for file

Additional file 2 **Table S2**. Gene annotation within the CNVRs retrieved from the Ensembl Genes 57 Database using the Biomart software. Click here for file

Additional file 3 **Fig. S1**. Results of quantitative PCR (qPCR) for CNVRs 15 (top), 17 (middle), and 32 (bottom). A total of 17 animals are showed in each plot. Breed abbreviations are: **Ib**: Iberian; **Ld**: Landrace; **Hib**: animals belonging to several generations of the IBMAP cross (F1, F2, and BC); **CC**: Cuban creole pig; **Gu**: Guatemala local breed; **Yu**: Yucatan miniature pig; **Pe**: Peruvian creole pig. Click here for file

Additional file 4 **Table S3**. List of pig genes previously reported in the Human Database of Genomic Variants. Click here for file

Additional file 5 **Fig. S2**. Structure of the IBMAP cross. Abbreviations are: **Ib**: Iberian; **Ld**: Landrace; **F1**: first generation; **F2**: second generation; **F3**: third generation; **BC**: first backcross; **BC1_LD**: second backcross. Click here for file

Additional file 6 **Table S4**. Description of samples from American local breeds. Click here for file

Additional file 7 **Table S5**. Primers and probes used in quantitative PCR validation Click here for file
