# Supplementary figures and images for: Copy number variation in the porcine genome inferred from a 60 k SNP BeadChip
Source: BMC Genomics. 2010 Oct 22;11:593. doi: 10.1186/1471-2164-11-593 (PMC3091738; doi:10.1186/1471-2164-11-593)

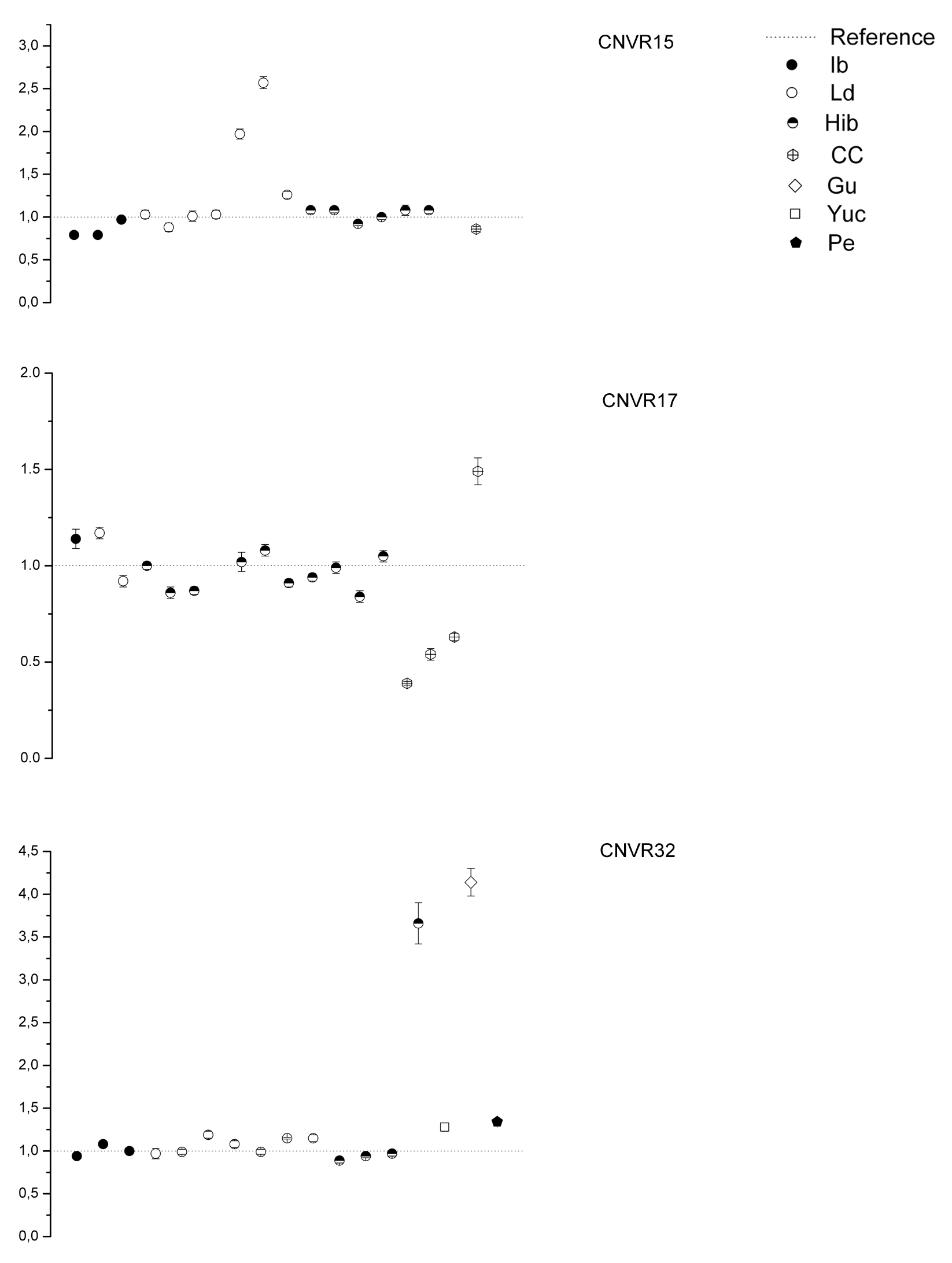

Supplement: Additional file 3 — Fig. S1. Results of quantitative PCR (qPCR) for CNVRs 15 (top), 17 (middle), and 32 (bottom). A total of 17 animals are showed in each plot. Breed abbreviations are: Ib: Iberian; Ld: Landrace; Hib: animals belonging to several generations of the IBMAP cross (F1, F2, and BC); CC: Cuban creole pig; Gu: Guatemala local breed; Yu: Yucatan miniature pig; Pe: Peruvian creole pig. [file 1471-2164-11-593-S3.TIFF]

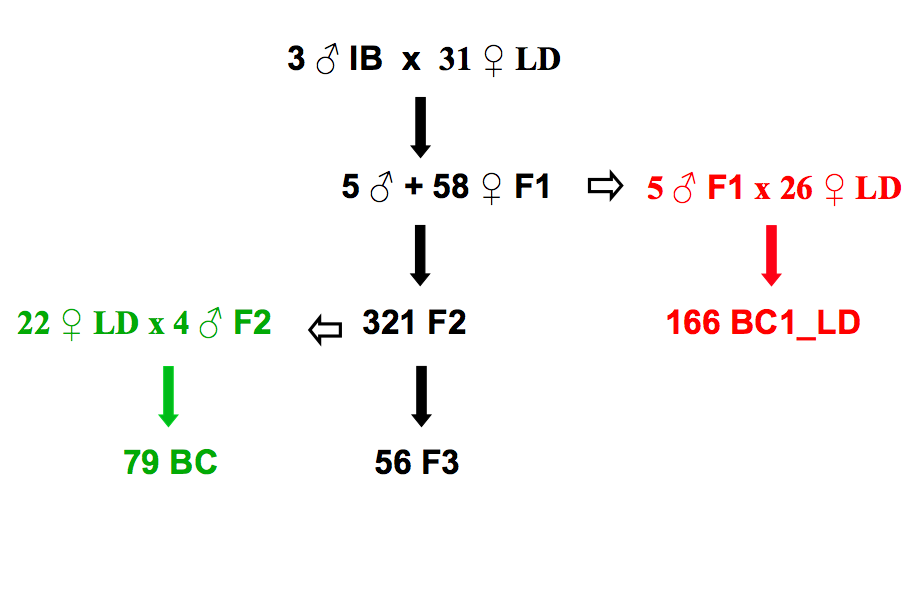

Supplement: Additional file 5 — Fig. S2. Structure of the IBMAP cross. Abbreviations are: Ib: Iberian; Ld: Landrace; F1: first generation; F2: second generation; F3: third generation; BC: first backcross; BC1_LD: second backcross. [file 1471-2164-11-593-S5.TIFF]
