# Supplementary material for: Copy number variation in the porcine genome inferred from a 60 k SNP BeadChip
Source: BMC Genomics. 2010 Oct 22;11:593. doi: 10.1186/1471-2164-11-593 (PMC3091738; doi:10.1186/1471-2164-11-593)
Supplement: Additional file 4 — Table S3. List of pig genes previously reported in the Human Database of Genomic Variants. [file 1471-2164-11-593-S4.DOC]

**Additional file 4, Table S3:** List of pig genes previously reported in the Human Database of Genomic Variants.

| **Pig Gene** | **ID Human Database**^*^ |
| --- | --- |
| MANEA | Variation_59367 |
| FUT9 | Variation_5225 |
| KIAA0776 | Variation_67124 (KIAA07) |
| KLHL32 | Variation_22653 |
| POU3F2 | Variation_81507 |
| FBXL4 | Variation_64603 |
| MCHR2 | Variation_8525 |
| SIM1 | Variation_51772 – 51774 |
| GRIK2 | Variation_1135-1126, 7511,23778, 32829, 36442, 81514, 93918 |
| FRK | Variation_93972 |
| DSE | Variation_99676 |
| LGSN | Variation_3617 |
| ACAA2 | Variation_49999 |
| CCDC102B | Variation_29680 |
| PTPRD | Variation_1470 |
| GLDC | Variation_3757 |
| UHRF2 | Variation_100571 |
| RANBP6 | Variation_70512 |
| KIAA1432 | Variation_44391 |
| CD274 | Variation_52834 |
| C9orf46 | Variation_2787 |
| JAK2 | Variation_1812 |
| AK3 | Variation_70511 |
| CDC37L1 | Variation_59534 |
| C9orf68 | Variation_65315 |
| SLC1A1 | Variation_2785 |
| RFX3 | Variation_100561 |
| KIAA0020 | Variation_100559 |
| SMARCA2 | Variation_38738 |
| DMRT2 | Variation_3755 |
| KANK1 | Variation_0126 |
| DMRT1 | Variation_22531 |
| PGM5 | Variation_31516 |
| FAM122A | Variation_5262 |
| FXN | Variation_100650 |
| TJP2 | Variation_22576 |
| SERPINI1 | Variation_0039 |
| ZP4 | Variation_2353 |
| RYR2 | Variation_30462 |
| FAM117B | Variation_98108 |
| ALS2CR8 | Variation_6021 |
| INSC | Variation_48751 |
| PDE3B | Variation_23403 |
| CNBD1 | Variation_5257 |
| SLC16A7 | Variation_66240 |
| TRHDE | Variation_3895 |
| FUT8 | Variation_37161 |

### ^*^ <http://projects.tcag.ca/variation/?source=hg19>
