# Supplementary material for: Copy number variation in the porcine genome inferred from a 60 k SNP BeadChip
Source: BMC Genomics. 2010 Oct 22;11:593. doi: 10.1186/1471-2164-11-593 (PMC3091738; doi:10.1186/1471-2164-11-593)
Supplement: Additional file 6 — Table S4. Description of samples from American local breeds. [file 1471-2164-11-593-S6.DOC]

### Additional file 6, Table S4: Description of samples from American local breeds.

| **Country** | **Breed** | **N** |
| --- | --- | --- |
| **USA** | Yucatán | 10 |
| **Mexico** | Hairless | 2 |
| **Cuba** | Creole | 18 |
| **Guatemala** | Creole | 14 |
| **Costa Rica** | Creole | 12 |
| **Peru** | Creole | 16 |
| **Argentina** | Creole / feral | 24 |
| **Brazil** | Moura | 9 |
|  | Nilo | 2 |
|  | Monteiro | 10 |
|  | Piau | 10 |
