# Supplementary material for: Copy number variation in the porcine genome inferred from a 60 k SNP BeadChip
Source: BMC Genomics. 2010 Oct 22;11:593. doi: 10.1186/1471-2164-11-593 (PMC3091738; doi:10.1186/1471-2164-11-593)
Supplement: Additional file 7 — Table S5. Primers and probes used in quantitative PCR validation [file 1471-2164-11-593-S7.DOC]

**Additional file 7, Table S5**: Primers and probes used in qPCR validation

| **Chr** | **CNVR** | **Gene or EST** | **Forward** | **Reverse** | **Taqman MGB Probe** |
| --- | --- | --- | --- | --- | --- |
| 5 | CNVR22 | *SLC16A7* | 5'-CCTGTGCTGGAGACCAGGTAA-3' | 5'-CCCGGGTAGCTCTGGTAACA-3' | 5'FAM-TCCCGGGGTGTAACT-3' |
| 14 | CNVR36 | *CYTP450 2C32* | 5'-TACAGTTAAATGTTAAGGACATCAGCAA-3' | 5'-TGCAACCCAAAGGGATGTAAA-3' | 5'FAM-TCCTTAAGCAACGTAAGTAT-3' |
| 2 | CNVR15 | *INSC* | 5'-CCACTTTGCAGACAGTAAACTTCTATTC-3' | 5'-TGGGAAGCTCCAGAGAGGAA-3' | 5'FAM-TCCTTGAAACCCAGCCAA-3' |
| 1 | CNVR1 | *NMBR* | 5´-TGTTCACTCTCACTGCCCTCAG-3 | 5'-CCATCGTCCTTTCTCCTTCCT-3' | 5'FAM-ACCTGTGCCCTTACCT-3' |
| 1 | CNVR3 | *EW037329 (EST)* | 5´-CTTATATAGCCAAAATGATCAATGTCAAG-3' | 5'-GTTGTGCTTTTTTTGTCTATATATGTTCCA-3' | 5'FAM-CCTGGTCTAGTAAAATT-3' |
| 13 | CNVR32 | *SOX14* | 5'-GCCTGGGTGCCGAGACT-3' | 5´-AGCCAGAGAGAAGCGCTAACC-3' | 5'FAM-CAGCTGAACTTTGCG-3' |
| 4 | CNVR17 | *DY435758 (EST)* | 5'-CATAAAACCACAATTCAGTGAATTGTC-3' | 5'-GCCATTTTGGTTTCACGTTAATC-3' | 5'FAM-ACTGTAATCTACAAAAGAGC-3' |
| 15 | Control | *GCG* | 5'-AACATTGCCAAACGTCACGATG-3' | 5'-GCCTTCCTCGGCCTTTCA-3' | 5'VIC-ACATGCTGAAGGGACC-3' |
